# An American Contribution to Bacteriology

**Published:** 1886-08

**Authors:** 


### DANIEL'S Medical Journal,

PUBLISHED MONTHLY AT

AUSTIN, TEXAS.

F. E. DANIEL, M. D., EDITOR AND PUBLISHER

Subscription Price: Two Dollars per Annum in Advance.

COLLABORATORS.

*E. J. Doering, M. D., Chicago.*

*Geo. Cupples, M. D.. San Antonio.*

*Odo Betz, M. D., Germany.*

*E. J. Beall, M. D., Fort Worth, Texas.*

*T. C Osborn, M D., Cleburne,* "

*Wm. Penny, M D., New York.*

*H. O. Marcy, M. D., Boston.*

*C. K. Gregg, M. D., Mexico.*

*R. M Swearingen, M. D.. Austin*

*C. H. Wilkinson, M. D., Galveston.*

*J. W. McLaughlin, M D., Austin.*

*R. H. L. Bibb, M. D., Mexico.*

### EDITORIAL.

### AN AMERICAN CONTRIBUTION TO BACTERIOLOGY.

Under the above caption the *New York Medical Record* speaks editorially, and quite at length, of the discovery, by Dr. McLaughlin of Austin, of the micrococcus of dengue.

This article, while complimentary in the main, is strangely contradictory. The *Record* describes with minuteness the details of Dr. McLaughlin's investigations and the result, praises the doctor for being sufficiently interested in science to find time from a laborious practice, to make such experiments; and commends the example to others who, tho' scientifically inclined, say they "have not time" to make experiments. (*En passant* we will say that the life-work of Gross, Flint and F. H. Hamilton should be suffic ent refutation of any such assertions —those eminent practitioners having found time from their professional duties to contribute very largely to the literature of medicine and surgery.)

After saying "He [Dr. McL.] examined, microscopically, fresh specimens [of the blood of dengue patients] as well as others that were dried and stained and sterilized. He also obtained pure cultures of a micro-organism and watched its growth in successive cultivations. He believes there is always in the blood of these patients a peculiar organism, having its own method of growth, and taking up, with especial avidity, a stain of methyl-blue dissolved in caustic potash. This organism is spherical in shape, and about 1-20 to 1-30 the diameter of a red blood-cell. The organisms grew well upon sterilized jelly, *always preserving their morphological character in successive cultures*, [italics ours.] All the experiments seemed to show that the blood of persons suffering from dengue contains these micro-organisms, and no other,"—the *Record* closes by the startling assertion that "Dr. McLaughlin's experiments, therefore, are not conclusive of anything yet;" and that "it cannot be said that this micrococcus is not identical with that found in other diseases." [!!!]

Here, we say, is a strange contradiction. According to the *Record's* admission, the researches prove: 1st, That dengue is a contageous disease and is produced by a living organism; 2nd, That this organism always preserves its morphological (and, therefore, distinguishing) character; 3d, That it has its own peculiar stain. And, according to Dr. McLaughlin's paper, the investigations prove, 4th, That micro-organisms can and do exist in the blood cells;—something, according to Friedlander, not heretofore demonstrated. The above would seem to be "conclusive" of something.

As to the identity with the organisms found in the blood of other diseases, the micro-coccus of dengue as shown by Dr. McL. differs materially, 1st. According to Friedlander, but two diseases are known to have micro-organisms uniformly found in the blood, viz: Anthrax, and Relapsing fever. In the first named we find a rod-shaped, and in the second a spiral-shaped bacteria *in the plasma, but not in the cell*; while that of dengue is spherical, and is found in the blood cell, as well as in the plasma. The micro-coccus of dengue therefore cannot be "identical with that of other diseases." 2nd. The coccus of dengue does not stain with analine dyes in the uniform manner which the organisms named, and most others, do.

The only other cocci *said* to be found in the blood cell are those of malarial fever as recently claimed by Levarron; and from these, the cocci of dengue are differentiated—3rd. By their morphological character, and their behavior with staining agents.

The *Record* in closing its comments says: "Dr. McLaughlin has come quite near being the first American physician to find the specific micro-organism of a disease affecting man."

We would ask the *Record* to please name the American physician who has been the first to find such organism? as the remark implies that the micro-organism has been found by an American physician and that Dr. McLauglin is not that physician! Or, to be generous, did the *Record* mean to say that Dr. McLaughlin was the first American physician who had come very near finding a specific organism?

*Apropos*, of the *Micro-coccus McLaughlinii* [Daniel,] we observe with some surprise that our usually just and courteous contemporary of the N. O. M. & S. Journal claims (August No.—page 147) to be the first to bring the subject of Dr. McLaughlin's researches and discovery to the notice of the profession, and we believe, cites the April Number of that Journal as containing the article. We beg to refer our friends of the N. O. M. & S. Journal and others, to the November Number of DANIEL'S TEXAS MEDICAL JOURNAL, (Vol. 1, No. V. p. 238), for a full description of Dr. McLaughlin's experiments, methods and discovery from reports to us by Dr. McLaughlin, and from ocular examinations of the specimens, which announcement we *know* to be the first made of the "interesting and important discovery."

The advice given to the man who had attempted to conceal a large fish under a short jacket was—"you should wear a longer jacket or steal a smaller fish" We suggest to our N. O. contemporary to read the TEXAS JOURNAL more closely, or to give us credit for some interest in the work of the profession of our State. We take this occasion, however, to thank our worthy contemporary for the kind and complimentary mention it has made of our friend and contributor Dr. McLaughlin and his works, in several numbers.

